# Cognitive complaints in body dysmorphic disorder: An exploratory characterization across clinical and community samples

**DOI:** 10.1017/S0033291726105030

**Published:** 2026-06-26

**Authors:** Katrina Holmes à Court, Tamsyn E. Van Rheenen, Susan Lee Rossell

**Affiliations:** 1 Centre for Mental Health and Brain Sciences, School of Health Sciences, Swinburne University of Technology, Hawthorn, Australia; 2 The University of Melbourne, Australia; 3 InsideOut Institute, University of Sydney and Sydney Local Health District, Sydney, Australia

**Keywords:** body dysmorphic disorder, cognitive complaints, processing speed, social cognition, self-efficacy, subjective cognition

## Abstract

**Background:**

Cognitive complaints in body dysmorphic disorder (BDD) remain poorly characterized. This study examined subjective cognitive complaints across clinical and community BDD samples.

**Methods:**

A two-stage exploratory design was used. Study 1 included a clinically diagnosed BDD sample (*n* = 15) and healthy controls (*n* = 29). Study 2 comprised a large international community sample reporting clinically significant BDD symptoms (*N* = 433). Participants completed *BodyThink*, an exploratory BDD-focused measure of subjective cognitive complaints. In Study 1, additional clinical and performance-based cognitive measures contextualized subjective reports.

**Results:**

Across both studies, endorsed items spanned multiple cognitive domains, with processing speed, attention, memory, executive functioning, and social cognition items consistently reported. Processing speed complaints were particularly salient. In Study 1, individuals with BDD reported markedly elevated cognitive complaints relative to controls, with large group differences (*d* = 1.64–1.95) on both *BodyThink* and an established measure of subjective cognition. Cognitive complaints showed a preliminary association with perceived social self-efficacy (*ρ* = −.71) but not with BDD symptom severity, objective cognitive performance, or emotional distress. Across both samples, social cognition items were disproportionately endorsed during symptom exacerbation.

**Conclusions:**

Individuals with BDD reported markedly elevated cognitive complaints relative to controls, with substantial individual variation. Patterns were broadly consistent across symptom severity, with social cognition difficulties showing greater salience during symptom exacerbation. The variability in complaints highlights the importance of individualized assessment, while associations with perceived social self-efficacy suggest that cognitive complaints may reflect negatively biased self-appraisals that may be relevant to treatment engagement.

## Introduction

Cognitive–behavioral models of body dysmorphic disorder (BDD) propose that symptoms develop and are maintained through dysfunctional self-referential processing, including biased interpretation of appearance-related feedback and negatively biased self-evaluation (Fang & Wilhelm, 2015; Veale, 2004a). Despite affecting ~2% of the population and causing substantial impairment across employment, education, and quality of life (Bjornsson, Didie, & Phillips, 2010; Phillips, Menard, Fay, & Pagano, 2005), BDD remains widely under-recognized in clinical settings (Rossell, 2023). The disorder is characterized by excessive preoccupation with perceived appearance defects and associated repetitive behaviors that cause clinically significant distress (American Psychiatric Association, 2013). BDD also commonly co-occurs with other psychiatric conditions, including major depressive disorder, anxiety disorders, and obsessive-compulsive disorder (Gunstad & Phillips, 2003).

Objective assessments of cognition in BDD have reported both global and domain-specific impairments, including in attention (Toh, Castle, & Rossell, 2017), processing speed ( Holmes à Court, Byrne, et al., 2026), memory (Deckersbach et al., 2000), executive function (Dunai et al., 2010), and social cognition (Buhlmann, Wacker, & Dziobek, 2015), although findings have not been consistently replicated. A recent systematic review of cognition in BDD ( Holmes à Court, Malcolm, Toh, & Rossell, 2025), informed by a hierarchical model of cognition (Harvey, 2019), highlighted that while impairments are reported across multiple domains, they are most consistently observed in higher-order domains such as executive function and social cognition.

However, objective cognitive testing alone cannot fully capture how individuals experience their thinking in everyday life (Toplak, West, & Stanovich, 2013; Van Patten et al., 2025). Subjective cognition – hereafter referred to as cognitive complaints – reflects individuals’ metacognitive appraisals of their cognitive functioning and may diverge from objectively measured performance. These complaints are clinically relevant because they can shape perceived competence, everyday functioning, and treatment engagement, even when they do not directly correspond to objective deficits. In psychiatric conditions related to BDD, cognitive complaints have been associated with functional impairment and psychological distress and, in some cases, predict aspects of objective performance, particularly in attention and processing speed (e.g. Arts, Jabben, Krabbendam, & van Os, 2011). Characterizing cognitive complaints in BDD, therefore, represents an important and underexplored component of the disorder’s cognitive profile.

Emerging evidence suggests that cognitive complaints are prominent in BDD. Two qualitative studies (Brennan et al., 2023; Holmes à Court, Van Rheenen, & Rossell, 2025) and one mixed-methods study ( Holmes à Court, Van Rheenen, & Rossell, 2026) indicate that individuals with BDD often describe their cognition as globally deficient or ‘fundamentally flawed’ (Brennan et al., 2023, p. 4), with persistent difficulties that affect daily functioning. However, no BDD-focused structured instrument has been used to characterize these complaints across multiple cognitive domains.

The present study aimed to quantitatively characterize cognitive complaints in BDD using a novel self-report instrument. Study 1 was a pilot investigation in a small, clinically diagnosed BDD sample and healthy controls, enabling initial comparison of cognitive complaints in a diagnostically well-characterized group. Study 2 extended this work to a larger international online sample of individuals who self-reported a BDD diagnosis or met a validated symptom threshold, allowing assessment of the consistency of complaint patterns in a broader community sample. We expected individuals with BDD in Study 1 to report elevated cognitive complaints relative to controls. In Study 2, we aimed to characterize the pattern and consistency of complaints in the community sample. Given the exploratory nature of this work and the unequal distribution of items across domains, no domain-specific hypotheses were specified.

## Method

### Study 1: Pilot clinical sample

Study 1 was nested within a clinical trial investigating intranasal oxytocin for BDD (ACTRN12622000123718) and uses baseline data collected before randomization.

#### Participants

The Study 1 sample comprised 15 individuals with a primary diagnosis of BDD and 29 controls. Participants were recruited via clinician referral and community advertising.

BDD diagnoses were established using the Body Dysmorphic Disorder Diagnostic Module for DSM-5 (BDD-DM; First, Williams, Karg, & Spitzer, 2015). Psychiatric comorbidity was assessed using the Mini-International Neuropsychiatric Interview (MINI-7; Sheehan et al., 1998). BDD participants were required to score ≥ 20 on the Yale–Brown Obsessive-Compulsive Scale modified for BDD (BDD-YBOCS; Phillips et al., 1997).

Exclusion criteria included current eating disorder (within 3 months), substance use disorder, neurological or uncontrolled medical illness, steroid use, oxytocin sensitivity, pregnancy, or breastfeeding. Control participants were excluded if they met criteria for any current or lifetime psychiatric disorder.

All participants were aged 18–65 years, fluent in English, and had an estimated premorbid IQ >70 (Test of Premorbid Functioning [TOPF]; Shura et al., 2022). Additional eligibility criteria included no uncorrected sensory impairments and, where applicable, stable psychotropic medication use for at least 8 weeks.

#### Procedure

Written informed consent was obtained from all participants. The study was approved by the Swinburne University Human Research Ethics Committee (SUHREC-5412). Following an initial telephone screening, participants attended a single in-person assessment session during which diagnostic status and symptom severity were confirmed, and clinical and social-cognitive tasks were administered. The *BodyThink* questionnaire was completed online via Qualtrics.

### Study 2: Online survey sample

#### Participants

Of 695 respondents, 433 met eligibility criteria and were included in the sample; the remaining respondents (*n* = 262) were excluded as they did not meet a conservative dysmorphic concern threshold (Dysmorphic Concern Questionnaire: DCQ < 14). Eligibility required endorsement of BDD symptoms and clinically significant dysmorphic concern (DCQ ≥ 14; Gieler et al., 2016; Mancuso, Knoesen, and Castle, 2010; Schieber, Kollei, de Zwaan, and Martin, 2018; Stangier et al., 2003), with diagnostic certainty varying across three subgroups: (1) formally diagnosed with BDD, (2) probable (i.e. participants who reported symptoms consistent with BDD and indicated uncertainty regarding whether they had received a formal clinical diagnosis), and (3) DCQ-defined cases (Table 1).

**Table 1.** Demographic, cognitive, and clinical characteristics of the Study 2 community sample Table 1. long description.

	BDD
	Mean (SD) or *n* (%)
*Demographic*	
Age	28.84 (9.80)
Age range	18–73
Sex, *n* = female	372 (85.9%)
Sex, *n* = male	54 (12.5%)
Sex, prefer to self-describe	7 (1.6%)
Years of education	15.83 (3.09)
*Ethnicity*	
Caucasian/White	316 (73.0%)
Asian	39 (9.0%)
Other	50 (11.5%)
African	14 (3.2%)
Hispanic	12 (2.8%)
Aboriginal/Torres Strait Islander	2 (0.5%)
*Clinical characteristics*	
DCQ	16.97 (2.11)
*Subjective cognitive complaints*	
*BodyThink*	65.07 (16.49)
*BodyThink by diagnostic category*	
Formally diagnosed *n* = 106	65.38 (16.81)
Probable *n* = 47	65.89 (20.07)
DCQ-defined *n* = 280	64.81 (15.76)

*Note: N* = 433. Diagnostic categories: Formally diagnosed = self-reported formal BDD diagnosis; Probable = reported symptoms consistent with BDD and indicated uncertainty regarding formal diagnosis; DCQ-defined = DCQ ≥ 14 without self-reported BDD. Abbreviations: DCQ, Dysmorphic Concern Questionnaire.

#### Design and procedure

Study 2 employed a cross-sectional online survey design. Ethical approval was granted by the Swinburne University Human Research Ethics Committee (20247235–17814), and all participants provided informed consent electronically before participation.

The survey was accessible internationally via Qualtrics between October 2023 and January 2025. Participants were recruited through social media platforms and email distribution lists supported by national and international body dysmorphic disorder and obsessive-compulsive disorder advocacy organizations. Participation was voluntary, and no financial compensation was provided.

### Measures

Across both studies, participants completed demographic questions. Study-specific measures are described below.

#### Cognitive complaints questionnaire (BodyThink)

To assess cognitive complaints in BDD, we developed *BodyThink*, a brief self-report questionnaire designed to capture everyday cognitive difficulties as described by individuals with BDD. As no disorder-specific measure of cognitive complaints currently exists for BDD, *BodyThink* was developed as an exploratory research instrument.

Items were informed by consultation with cognitive neuropsychiatry researchers and individuals with lived experience of BDD. They were adapted from established measures of cognitive complaints in psychiatric populations – the Cognitive Complaints in Bipolar Disorder Rating Assessment (COBRA; Rosa et al., 2013) and the Subjective Scale to Investigate Cognition in Schizophrenia (SSTICS; Stip et al., 2003) – and supplemented with novel items targeting BDD-relevant experiences (see Supplementary Table S1). These novel items target cognitive processes such as intrusive thought suppression (e.g. inhibiting recurring negative self-thoughts) and the interpretation of social-evaluative information (e.g. others’ intentions when receiving compliments). Such items were representative of cognitive complaints that lived-experience consultants identified with. Further detail on item development and rationale, including the cognitive/psychopathology boundary, is provided in Supplemental File A.

*BodyThink* assesses seven domains: processing speed, executive function, social cognition, language, attention, memory, and visuospatial. Items were unevenly distributed across domains due to partial adaptation from existing measures (COBRA and SSTICS) and the inclusion of selected BDD-specific items while minimizing participant burden. Consequently, domains were represented by differing numbers of items, precluding formal statistical comparison of domain scores. Analyses, therefore, focused on total scores and item-level patterns, with domain labels used descriptively to characterize which cognitive areas were represented among highly endorsed items. Participants rate the frequency of each difficulty on a four-point Likert scale (0 = *never*, 1 = *sometimes*, 2 = *often*, 3 = *always*), responding to the prompt ‘I generally….’ Item scores are summed to yield a total score (41 items; range 0–123), with higher scores indicating more frequent cognitive complaints.

To examine perceived symptom-linked exacerbation, participants subsequently re-rated the same items in response to the prompt, ‘Which of the thinking skills that you have just answered questions on are more noticeably difficult when your BDD symptoms are at their worst?’. Across both studies, analyses focused on total scores and item-level patterns derived from a common item set administered in each sample, enabling direct comparison of cognitive complaints.

#### Dysmorphic Concern Questionnaire

The Dysmorphic Concern Questionnaire (DCQ; Oosthuizen, Lambert, & Castle, 1998) is a seven-item self-report measure of dysmorphic concern (range: 0–21), with higher scores indicating greater severity. It has demonstrated good reliability and validity in clinical and community samples, and was used to index symptom severity and support participant classification in Study 2.

#### Cognitive Complaints in Bipolar Disorder Rating Assessment (COBRA; Study 1 only)

COBRA (Rosa et al., 2013) is a 16-item self-report measure of cognitive complaints across attention, memory, executive function, and processing speed (range: 0–48). It was included in Study 1 to contextualize cognitive complaints using an established instrument.

#### Additional Clinical and Cognitive Measures (Study 1 only)

Study 1 participants completed measures of BDD severity (BDD-YBOCS; Phillips et al., 1997), insight (BABS; Eisen et al., 1998), social self-efficacy (PSSE; Smith & Betz, 2000), and psychological distress (DASS-21; Lovibond & Lovibond, 1995). Performance-based assessments included social cognition (BLERT [Bell, Bryson, and Lysaker, 1997]; Hinting Task [Corcoran, Mercer, and Frith, 1995]; and TASIT-S [McDonald, Flanagan, & Rollins, 2006]) and processing speed (WAIS-IV PSI [Wechsler, 2008]).

### Data analysis

Analyses were conducted in R (R Core Team, 2022). All tests were two-tailed with *α* = .05. In Study 1, group differences were examined using Welch’s *t*-tests for continuous variables and Fisher’s exact tests for categorical variables; Mann–Whitney *U* tests were applied where assumptions were violated. Effect sizes (Cohen’s *d*) and 95% confidence intervals are reported for all group comparisons. For Mann–Whitney *U* tests, *d* was derived from the rank-biserial correlation, with confidence intervals estimated using bias-corrected and accelerated (BCa) bootstrap (2,000 replications). In Study 2, participants completing ≥95% of items were included; remaining missing data were handled using pairwise deletion. Differences in *BodyThink* total scores across the three diagnostic-certainty subgroups (formal/probable/DCQ-defined) were examined using Welch’s one-way ANOVA, consistent with the variance-equal-not-assumed approach used elsewhere in the paper, with omega-squared (*ω* ^2^) reported as the effect size. Across both studies, Spearman correlations examined associations between *BodyThink* scores and clinical measures.

## Results

Results are presented separately for the pilot clinical sample (Study 1) and the community sample (Study 2), followed by cross-sample comparisons.

### Study 1

#### Sample characteristics

Demographic and clinical characteristics are presented in Table 2. The BDD (*n* = 15) and control (*n* = 29) groups did not differ significantly in age, sex, years of education, or intellectual functioning (TOPF; all *p*s > .13). As expected, the BDD group reported significantly greater symptom severity (BDD-YBOCS), poorer insight (BABS), higher dysmorphic concern (DCQ), and greater overall psychological distress (DASS-21) relative to controls (all *p*s < .001). BDD participants reported a mean of 3.54 (1.76) comorbid psychiatric diagnoses (Table 2), consistent with established BDD comorbidity profiles (Gunstad & Phillips, 2003).

**Table 2.** Demographic, cognitive, and clinical characteristics of the Study 1 pilot sample Table 2. long description.

	BDD, *n* = 15	HC, *n* = 29			
	Mean (SD) or *n* (%)	Mean (SD) or *n* (%)	Test statistic	*p*	Effect size [95% CI]
*Demographic*					
Age†	36.15 (11.22)	33.55 (13.90)	*t*(28.44) = 0.64	.525	0.20 [−0.46, 0.85]
Sex, *n* = female	11 (73.3%)	20 (69.0%)	Fisher’s *p*	.453	φ = .16 [−.15, .45]
Years of education†	15.69 (2.90)	17.19 (2.85)	*t*(22.81) = −1.56	.133	−0.52 [−1.18, 0.14]
ToPF†	114.54 (8.86)	112.97 (10.52)	*t*(27.29) = 0.50	.620	0.16 [−0.50, 0.81]
Caucasian/White, *n* (%)	14 (93.3%)	24 (82.8%)	Fisher’s *p*	.647	φ = .15 [−.16, .42]
*Clinical characteristics*					
BDD-YBOCS	30.38 (5.42)	0.00 (0.00)	*t*(12) = 20.20	<.001	10.23 [7.89, 12.55]
DCQ‡	18.20 (4.51)	8.32 (2.02)	*t*(17.07) = 8.07	<.001	3.18 [2.25, 4.11]
BABS†	13.53 (4.04)	0.21 (1.11)	*t*(12.82) = 11.69	<.001	5.55 [4.16, 6.91]
DASS–21†	49.23 (21.35)	13.66 (10.77)	*t*(14.81) = 5.69	<.001	2.41 [1.56, 3.24]
PSSE†	59.15 (17.03)	—			
Number of comorbid psychiatric diagnoses	3.54 (1.76)	*n*/a			
Psychotropic medication use, *n* (%)	6 (46.2%)	0 (0.0%)	Fisher’s *p*	<.001	φ = .61 [.38, .77]
*Cognitive measures*					
BLERT†	84.26 (9.78)	85.22 (10.36)	*t*(24.44) = −0.29	.775	−0.09 [−0.75, 0.56]
Hinting task†	81.33 (7.63)	83.75 (4.83)	*t*(16.47) = −1.05	.308	−0.42 [−1.07, 0.25]
TASIT-S†	80.77 (7.60)	80.34 (14.76)	*t*(39.05) = 0.12	.903	0.03 [−0.62, 0.69]
WAIS-IV PSI†	93.85 (16.20)	101.83 (22.52)	*t*(31.63) = −1.30	.203	−0.38 [−1.04, 0.28]

*Note:* †*n* = 13 BDD due to missing data; ‡*n* = 28 HC due to missing data; PSSE was not administered to healthy controls. Effect sizes are Cohen’s *d* for continuous variables and *φ* (phi) for 2 × 2 categorical variables. Abbreviations: BABS, Brown Assessment of Beliefs Scale; BDD-YBOCS, Body Dysmorphic Disorder version of the Yale-Brown Obsessive Compulsive Scale; BLERT, Bell Lysaker Emotion Recognition Task; DASS-21, Depression Anxiety Stress Scales – 21 item version; DCQ, Dysmorphic Concern Questionnaire; PSSE, Perceived Social Self-Efficacy; TASIT-S, The Awareness of Social Inference Test – Short Form; ToPF, test of premorbid functioning; WAIS-IV PSI, Wechsler Adult Intelligence Scale – Fourth Edition Processing Speed Index. Group differences were analyzed using Welch’s *t*-tests (*t*) for continuous variables and Fisher’s Exact tests for categorical variables. *p < .05; **p < .01; ***p < .001 (two-tailed significance).

#### Group differences in cognitive complaints

Participants with BDD reported significantly greater cognitive complaints than control participants on *BodyThink* (BDD: *M* = 61.93, SD = 27.94, range 9–102; HC: *M* = 14.17, SD = 26.72, range 0–81), with a very large group difference (Mann–Whitney *U*, *p* < .001, 1.95, 95% CI: [1.30, 5.30]). Internal consistency was high in both the BDD (*α* = .94) and control (*α* = .98) groups.

To contextualize this finding, group differences were also examined on the COBRA, an established measure of cognitive complaints. Participants with BDD reported substantially higher complaints than controls (BDD: *M* = 24.67, SD = 9.43, range 6–40; HC: *M* = 6.93, *SD* = 13.06, range 0–45; 1.64, 95% CI: [1.05, 4.00], *p* < .001).

#### Item-level patterns of cognitive difficulties

**Figure 1.**
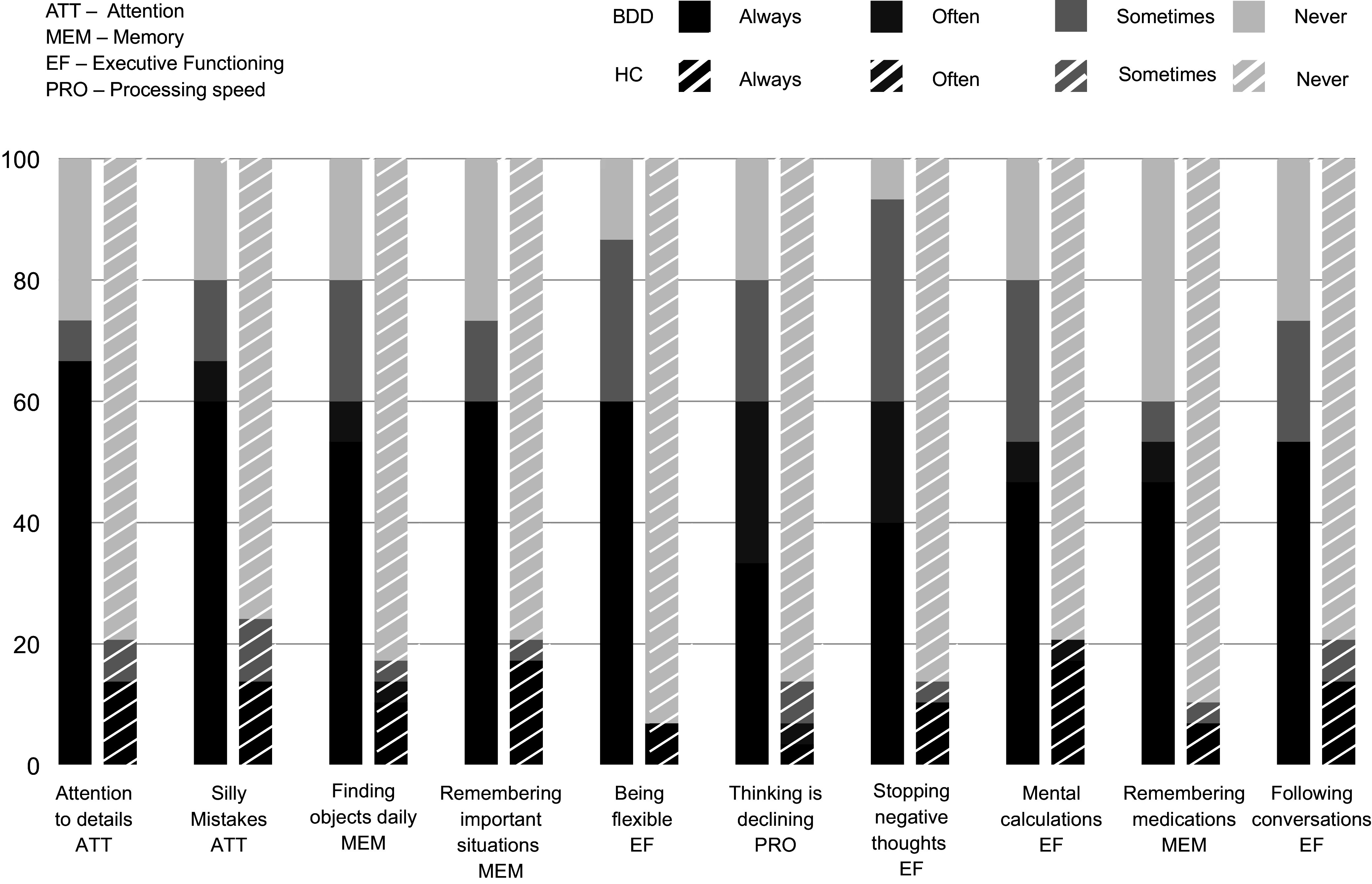
Top 10 endorsements of BodyThink items in the pilot sample. Figure 1. long description.

Item-level endorsement patterns on *BodyThink* were examined to characterize the nature and intensity of cognitive difficulties in the pilot BDD sample. Figure 1 presents stacked bar plots for the 10 most frequently endorsed items, shown separately for BDD and control participants. Individuals with BDD endorsed items spanning memory, processing speed, attention, and social cognition domains, whereas endorsement was minimal in controls. Processing speed items were most frequently endorsed as ‘always’.

#### Associations with clinical and objective cognition measures

Within the BDD group, higher *BodyThink* total scores were significantly associated with lower social self-efficacy (PSSE; *ρ* = −.71, 95% CI: [−.93, −.31], *p* = .007). No significant associations were observed between *BodyThink* and BDD symptom severity (BDD-YBOCS; *ρ* = .05, 95% CI: [−.50, .57], *p* = .869), symptoms of depression, anxiety, or stress as measured by the DASS-21 (all *p*s > .69), insight as measured by the BABS, objective processing speed as indexed by the WAIS-PSI, or social inference performance on the TASIT-S Part 2/3 Minimal. Given the exploratory nature of these analyses and the small sample size, no correction for multiple comparisons was applied.

### Study 2

#### Sample characteristics

Demographic characteristics of the Study 2 community sample (*N* = 433) are presented in Table 1. *BodyThink* scores did not significantly differ by diagnostic certainty (Welch’s *F*(2, 108.08) = 0.09, *p* = .912); analyses therefore used the combined sample. DCQ internal consistency was acceptable (*α* = .75), and scores ranged from 14 to 21 (*M* = 16.97, SD = 2.11), consistent with clinically significant dysmorphic concern.

#### Cognitive difficulties in the community sample

Participants reported frequent cognitive difficulties on *BodyThink* (*M* = 65.07, SD = 16.49, range 10–112). Internal consistency was acceptable (*α* = .81).

#### Item-level patterns and clinical correlates

**Figure 2.**
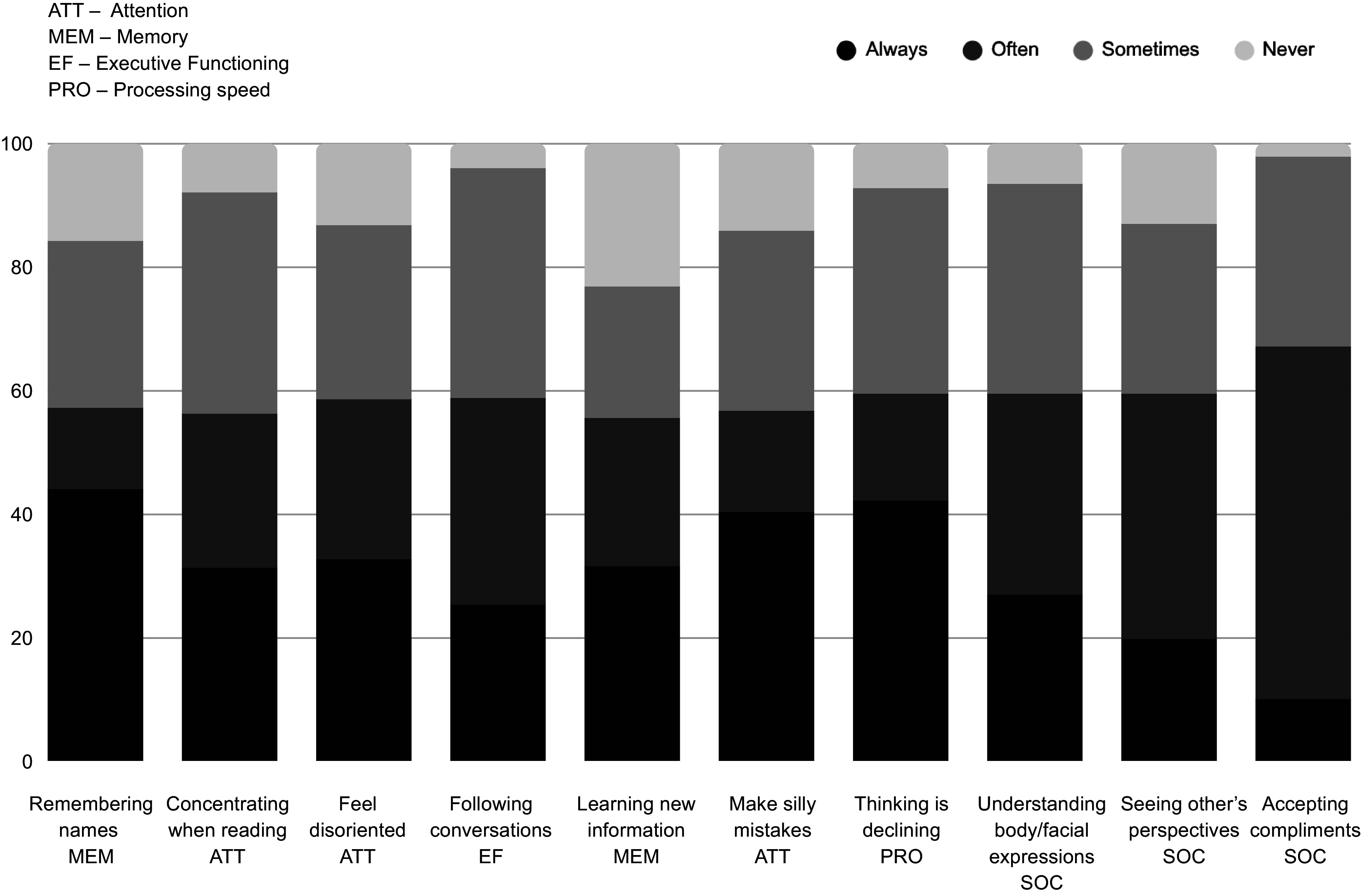
Top 10 endorsed BodyThink items in the community sample. Figure 2. long description.

Item-level endorsement patterns were examined to characterize cognitive difficulties in the community sample. Figure 2 presents stacked bar plots for the 10 most frequently endorsed *BodyThink* items. The most frequently endorsed items were drawn from the social cognition, attention, executive function, processing speed, and memory domains, with many participants endorsing these as occurring ‘often’ or ‘always’, indicating that cognitive complaints were both common and persistent.

Overall *BodyThink* scores were not associated with dysmorphic concern severity (DCQ; *ρ* = .06, 95% CI: [−.05, .15], *p* = .250), although interpretation is constrained by restricted variance due to the inclusion criterion (DCQ ≥ 14). *BodyThink* scores also did not differ by diagnostic certainty group (Welch’s *F*(2, 108.08) = 0.09, *p* = .912, *ω* ^2^ = .00); analyses are therefore presented for the combined sample.

### Cross-sample analyses

#### Convergence of item-level endorsement patterns across samples

Item-level endorsement rankings showed a moderate positive association between Study 1 and Study 2 (*ρ* .46, 95% CI: [.16, .69], *p* = .003), with four shared items among the 10 most frequently endorsed (see Figures 1 and 2). Despite limited item-level overlap, highly endorsed items in both samples were drawn from similar domains (memory, attention, executive functioning, and processing speed), with social cognition items more frequently endorsed in Study 2.

#### Symptom exacerbation ratings

When participants identified difficulties that were more noticeable during symptom exacerbation, endorsement patterns shifted in both samples. In Study 1, the most frequently endorsed exacerbation items were drawn primarily from social cognition (e.g. ‘recognizing people’, ‘taking things too literally’, and ‘understanding sarcasm’) and attention, with only 2 of 10 items overlapping with the general *BodyThink* top 10. In Study 2, social cognition items similarly became more prominent during exacerbation (e.g. ‘accurately reading social situations’, and ‘taking things too literally’), with 3 of 10 items overlapping with general ratings. Across both samples, social cognition items were more frequently endorsed during symptom exacerbation compared to general frequency ratings, suggesting that interpersonal cognitive difficulties may intensify when BDD symptoms are at their worst.

## Discussion

This study characterized cognitive complaints in BDD across a pilot clinical sample with healthy controls and a large international community sample. The BDD group reported substantially elevated cognitive complaints relative to controls on both the COBRA (1.64, 95% CI: [1.05, 4.00]) and *BodyThink* (1.95, 95% CI: [1.30, 5.30]), although scores varied markedly between individuals. Across both studies, endorsed *BodyThink* items spanned processing speed, attention, memory, executive functioning, and social cognition, with partial item-level overlap despite differences in sample composition and design. *BodyThink* extends existing measures by capturing BDD-relevant domains, including social cognition, which was prominently endorsed in the community sample.

Processing speed complaints were particularly prominent across both samples, aligning with evidence of processing speed deficits in BDD ( Holmes à Court, Byrne, et al., 2026) while leaving open whether subjective complaints reflect objective impairment, functional consequences, or metacognitive appraisal.

The observed pattern of complaints is broadly consistent with a hierarchical model of cognition, in which lower-level difficulties in processing speed, attention, and memory co-occur with higher-order difficulties in executive functioning and social cognition. Some *BodyThink* items, such as ‘difficulty accepting compliments’ and ‘stopping negative thoughts’, sit closer to the boundary between cognitive and psychopathological complaints than items assessing concentration, memory, or decision-making. However, these experiences remain cognitively mediated: ‘difficulty accepting compliments’ may involve the interpretation of social-evaluative information (others’ intentions and self-relevant evaluations), while ‘stopping negative thoughts’ may involve intrusive thought suppression and cognitive control. We therefore interpret these items as capturing subjective cognitive experiences closely embedded within core BDD phenomenology. Endorsement of these items is consistent with qualitative accounts of shame-based self-appraisal, fear of negative evaluation, and intrusive thought patterns described by individuals with BDD (Holmes à Court, Van Rheenen, et al., 2025).

In the pilot sample, cognitive complaints were independent of emotional distress, insight, and BDD symptom severity (BDD-YBOCS), but were associated with perceived social self-efficacy. Cognitive complaints were not significantly associated with processing speed or social cognition. These subjective–objective comparisons are preliminary, given the small sample, but tentatively suggest that perceived cognitive difficulties may not directly reflect objective impairment, and may instead reflect disorder-specific and interpersonal factors, including self-appraisal – consistent with the broader subjective cognition literature in which self-reported and objective cognition are recognized as related but distinct (Van Patten et al., 2025). The preliminary association with perceived social self-efficacy is consistent with the view that cognitive complaints may be embedded within broader negative self-schemas, in line with cognitive–behavioral models of biased self-referential processing. Such dissociations between subjective and objective cognition, and their association with self-appraisal and distress, are documented across mood, anxiety, and psychotic disorders (Van Patten et al., 2025); the present findings characterize this phenomenon as it presents in BDD.

BDD symptom severity (BDD-YBOCS) was not associated with cognitive complaints in the pilot sample, and a convergent pattern was observed in the community sample, where *BodyThink* scores were not associated with the severity of dysmorphic concern (DCQ). This cross-sample convergence suggests cognitive complaints persist across symptom severity levels, aligning with evidence that objective cognitive performance is similarly severity-independent in BDD ( Holmes à Court, Byrne, et al., 2026; Malcolm et al., 2021).

Item-level exacerbation ratings revealed a more nuanced picture. When asked about difficulties that intensify during symptom flares, participants in both samples disproportionately endorsed social cognition items (e.g. ‘recognizing people’, ;understanding sarcasm’, and ‘accurately reading social situations’), with minimal overlap between exacerbation-specific and general *BodyThink* rankings (2–3 of 10 items shared). Importantly, this pattern does not contradict the absence of association with BDD-YBOCS: symptom severity captures between-person differences in current symptom state, whereas exacerbation ratings capture within-person fluctuations relative to individual baseline. The selective endorsement of social cognition items during exacerbation suggests that these difficulties become more *salient* – rather than objectively worse – in social-evaluative contexts during symptom flares, when appearance-based scrutiny and interpersonal evaluation are heightened (Fang & Wilhelm, 2015). Cognitive experience in BDD may therefore involve broader cognitive self-appraisals that are not reducible to symptom severity, alongside heightened salience of interpersonal cognitive difficulties during social-evaluative symptom flares.

### Clinical implications

The present findings have three implications for BDD assessment and treatment. First, recognition – subjective cognitive complaints in BDD may be overlooked in routine practice. In a recent mixed-methods self-report study (Holmes à Court, Van Rheenen, et al., 2026), only 35% of participants reporting cognitive difficulties had discussed them with their treating team, 67% had not been offered any education about cognitive functioning, and many feared their concerns would not be taken seriously. *BodyThink* offers a brief, BDD-focused instrument to systematically probe these concerns during assessment.

Second, individualized formulation – the wide variation in *BodyThink* scores (10–112 on a 123-point scale in Study 2) parallels evidence of objective cognitive heterogeneity in BDD, where subgroup analyses have identified distinct cognitive profiles (Malcolm et al., 2021). Together, these findings indicate that cognitive experience differs markedly between individuals with BDD, supporting individualized case formulation.

Third, treatment engagement – the preliminary Study 1 association between cognitive complaints and perceived social self-efficacy, to be interpreted cautiously given the small sample, suggests that reduced perceived social self-efficacy may be relevant to engagement with cognitive–behavioral interventions involving social exposure (Rukmini, Sudhir, Bhaskar, & Arumugham, 2021), warranting attention in treatment planning pending replication. The increased prominence of social cognition difficulties during symptom exacerbation similarly indicates that interpersonal tasks central to exposure and response prevention (e.g. reducing camouflage and initiating eye contact) may require additional scaffolding.

### Limitations and future research

*BodyThink* is in the early phase of development. As an exploratory instrument, the current study was not designed to provide full psychometric validation, and further work is required to examine factor structure, test–retest reliability, and sensitivity to change. The small pilot clinical sample limited statistical power for correlational analyses, necessitating cautious interpretation.

Although shared method variance may have influenced associations among self-report measures, the absence of correspondence with objective cognition in Study 1 is preliminary, given the small sample; larger studies are needed to test whether the construct is distinct from simple reporting bias. The community sample relied on self-reported diagnosis and did not include a control group, restricting group-level specificity, although online recruitment in BDD has practical advantages, given that appearance-related shame can inhibit clinic attendance. Self-selection into a study focused on cognitive experiences in BDD may also have over-represented individuals who themselves perceive cognitive difficulties, biasing the Study 2 sample toward elevated complaints relative to the BDD population as a whole. The high proportion of women in both samples may limit generalizability, given gender differences in cognitive complaints in related disorders (Lin et al., 2019). The cross-sectional design also precludes conclusions about temporal relationships between symptom exacerbation, self-efficacy beliefs, and cognitive experience. The present findings should therefore be read as characterizing the phenomenon of subjective cognitive complaints in BDD without determining what these complaints reflect (e.g. genuine cognitive impairment, negative self-appraisal, distress, or broader psychopathology).

Future research should examine the longitudinal dynamics of cognitive complaints in BDD, including whether social cognition difficulties intensify during symptom flares and whether subjective cognition predicts CBT engagement or functional outcomes. Psychometric refinement should clarify *BodyThink*’s factor structure, sensitivity to change, and the position of items closer to the cognitive/psychopathology boundary (e.g. ‘difficulty accepting compliments’ and ‘stopping negative thoughts’). Larger studies should also test whether high versus low *BodyThink* scores identify clinically meaningful subgroups. Integrating subjective cognitive measures with objective tasks, ecological assessment, and clinician ratings may help disentangle perceived cognitive difficulties from objective performance, mood, distress, and broader psychopathology.

### Conclusion

These findings provide converging evidence that BDD is associated with elevated subjective cognitive difficulties across multiple domains. By introducing *BodyThink* as a brief self-report measure of subjective cognitive experience in BDD, this study highlights the clinical relevance of assessing cognitive self-appraisals alongside traditional symptom measures. These complaints may involve broader cognitive self-appraisals not reducible to symptom severity, alongside heightened salience of interpersonal cognitive difficulties during social-evaluative symptom flares. These findings have implications for formulation, treatment engagement, and functional outcomes.

## Supporting information

10.1017/S0033291726105030.sm001 Holmes à Court et al. supplementary material Holmes à Court et al. supplementary material

## Supplementary material

The supplementary material for this article can be found at http://doi.org/10.1017/S0033291726105030.

## Long descriptions

### Table 1. Long description

The table presents data for a B D D group, reporting Mean with Standard Deviation in parentheses or count with percentage in parentheses.

Demographic section:

* Age: 28.84 (9.80) with a range of 18 to 73.

* Sex: 372 females (85.9 percent), 54 males (12.5 percent), and 7 who prefer to self-describe (1.6 percent).

* Years of education: 15.83 (3.09).

Ethnicity section:

* Caucasian or White: 316 (73.0 percent).

* Asian: 39 (9.0 percent).

* Other: 50 (11.5 percent).

* African: 14 (3.2 percent).

* Hispanic: 12 (2.8 percent).

* Aboriginal or Torres Strait Islander: 2 (0.5 percent).

Clinical and Cognitive section:

* D C Q score: 16.97 (2.11).

* BodyThink score: 65.07 (16.49).

BodyThink scores by diagnostic category:

* Formally diagnosed (n equals 106): 65.38 (16.81).

* Probable (n equals 47): 65.89 (20.07).

* D C Q-defined (n equals 280): 64.81 (15.76).

Navigate back to Table 1..

### Table 2. Long description

The table consists of six columns: Variable, B D D n equals 15 Mean S D or n percentage, H C n equals 29 Mean S D or n percentage, Test statistic, p, and Effect size 95 percent C I.

Demographic section:

- Age: B D D 36.15 11.22, H C 33.55 13.90, t 28.44 equals 0.64, p equals .525.

- Sex female: B D D 11 73.3 percent, H C 20 69.0 percent, Fisher p equals .453.

- Years of education: B D D 15.69 2.90, H C 17.19 2.85, t 22.81 equals negative 1.56, p equals .133.

- T o P F: B D D 114.54 8.86, H C 112.97 10.52, t 27.29 equals 0.50, p equals .620.

- Caucasian White: B D D 14 93.3 percent, H C 24 82.8 percent, Fisher p equals .647.

Clinical characteristics section:

- B D D Y B O C S: B D D 30.38 5.42, H C 0.00 0.00, t 12 equals 20.20, p is less than .001.

- D C Q: B D D 18.20 4.51, H C 8.32 2.02, t 17.07 equals 8.07, p is less than .001.

- B A B S: B D D 13.53 4.04, H C 0.21 1.11, t 12.82 equals 11.69, p is less than .001.

- D A S S 21: B D D 49.23 21.35, H C 13.66 10.77, t 14.81 equals 5.69, p is less than .001.

- P S S E: B D D 59.15 17.03, H C not applicable.

- Comorbid diagnoses: B D D 3.54 1.76, H C not applicable.

- Psychotropic medication use: B D D 6 46.2 percent, H C 0 0.0 percent, Fisher p is less than .001.

Cognitive measures section:

- B L E R T: B D D 84.26 9.78, H C 85.22 10.36, t 24.44 equals negative 0.29, p equals .775.

- Hinting task: B D D 81.33 7.63, H C 83.75 4.83, t 16.47 equals negative 1.05, p equals .308.

- T A S I T S: B D D 80.77 7.60, H C 80.34 14.76, t 39.05 equals 0.12, p equals .903.

- W A I S I V P S I: B D D 93.85 16.20, H C 101.83 22.52, t 31.63 equals negative 1.30, p equals .203.

Navigate back to Table 2..

### Figure 1. Long description

A stacked bar chart displays ten items on the x-axis, each with two bars: a solid bar for B D D and a striped bar for H C. The y-axis represents percentage from 0 to 100.

Legend and Key:

* A T T stands for Attention.

* M E M stands for Memory.

* E F stands for Executive Functioning.

* P R O stands for Processing speed.

* B D D group uses solid colors: black for Always, dark gray for Often, medium gray for Sometimes, and light gray for Never.

* H C group uses striped patterns: black for Always, dark gray for Often, medium gray for Sometimes, and light gray for Never.

Data Trends:

Across all ten items, the B D D group shows significantly higher combined Always and Often endorsements compared to the H C group.

1. Attention to details A T T: B D D is approximately 65 percent Always and 5 percent Often. H C is approximately 15 percent Always and 5 percent Often.

2. Silly Mistakes A T T: B D D is 60 percent Always and 5 percent Often. H C is 15 percent Always and 5 percent Often.

3. Finding objects daily M E M: B D D is 55 percent Always and 5 percent Often. H C is 12 percent Always and 3 percent Often.

4. Remembering important situations M E M: B D D is 60 percent Always and 0 percent Often. H C is 18 percent Always and 2 percent Often.

5. Being flexible E F: B D D is 60 percent Always and 0 percent Often. H C is 5 percent Always and 2 percent Often.

6. Thinking is declining P R O: B D D is 35 percent Always and 25 percent Often. H C is 3 percent Always and 2 percent Often.

7. Stopping negative thoughts E F: B D D is 40 percent Always and 20 percent Often. H C is 10 percent Always and 2 percent Often.

8. Mental calculations E F: B D D is 45 percent Always and 8 percent Often. H C is 18 percent Always and 3 percent Often.

9. Remembering medications M E M: B D D is 45 percent Always and 8 percent Often. H C is 5 percent Always and 2 percent Often.

10. Following conversations E F: B D D is 55 percent Always and 0 percent Often. H C is 15 percent Always and 5 percent Often.

Navigate back to Figure 1..

### Figure 2. Long description

A stacked bar chart with a vertical y-axis ranging from 0 to 100 and a horizontal x-axis listing ten items. A legend at the top right defines four frequency categories from darkest to lightest: Always, Often, Sometimes, and Never. A key at the top left defines initialisms: A T T for Attention, M E M for Memory, E F for Executive Functioning, and P R O for Processing speed. S O C is also used for Social.

From left to right, the items and their approximate stacked percentages are:

1. Remembering names M E M: Always 44, Often 13, Sometimes 27, Never 16.

2. Concentrating when reading A T T: Always 31, Often 25, Sometimes 36, Never 8.

3. Feel disoriented A T T: Always 33, Often 26, Sometimes 28, Never 13.

4. Following conversations E F: Always 25, Often 34, Sometimes 37, Never 4.

5. Learning new information M E M: Always 32, Often 24, Sometimes 21, Never 23.

6. Make silly mistakes A T T: Always 40, Often 17, Sometimes 29, Never 14.

7. Thinking is declining P R O: Always 42, Often 17, Sometimes 33, Never 8.

8. Understanding body/facial expressions S O C: Always 27, Often 32, Sometimes 34, Never 7.

9. Seeing other's perspectives S O C: Always 20, Often 40, Sometimes 27, Never 13.

10. Accepting compliments S O C: Always 10, Often 57, Sometimes 31, Never 2.

Navigate back to Figure 2..
