# Supplementary material for: Cognitive complaints in body dysmorphic disorder: An exploratory characterization across clinical and community samples
Source: Psychol Med. 2026 Jun 26;56:e209. doi: 10.1017/S0033291726105030 (PMC13319485; doi:10.1017/S0033291726105030)
Supplement: Holmes à Court et al. supplementary material [file S0033291726105030sup001.docx]

**Supplemental File A**

***BodyThink* Item Development and Rationale**

*BodyThink* was developed in close consultation with people with lived experience of BDD, who associated BDD-relevant content (e.g., receiving compliments, intrusive negative self-thoughts) with their cognitive interference, that is, the rumination, distractibility, and difficulty disengaging that disrupts daily function. We therefore included items reflecting cognitive function in the contexts where it is most acutely impaired in BDD, rather than restricting items to symptom-neutral content that could underestimate the cognitive load these individuals experience.

Two of the novel items sit at the boundary between cognitive complaints and BDD psychopathology, where the distinction is not discrete. Both items nonetheless engage cognitive processes: *stopping negative thoughts* engages intrusive thought suppression, while *accepting compliments* engages the interpretation of social-evaluative information (others' intentions and self-relevant evaluations). We interpret these items as capturing cognitively mediated experiences embedded within BDD phenomenology rather than separable neurocognitive deficits, and note that future psychometric refinement is warranted.

Free-text responses from our prior mixed-methods study of self-reported cognition in BDD (Holmes à Court, Van Rheenen, & Rossell, 2026b) reinforce that participants themselves distinguish cognitive complaints from broader psychopathology, even where this content overlaps. Asked why they thought their cognitive difficulties were related to BDD, and what else they might be related to, some respondents distinguished BDD-related cognitive complaints from co-occurring non-cognitive symptoms. One labelled a section of their response *"Non thinking difficulties (psych ones)"* and listed mood swings, self-criticism, and self-hatred separately from cognitive complaints such as executive paralysis, slowed processing, and unpredictable memory. Another framed intrusive thoughts as a cognitive resource drain rather than as the disorder itself: *"the extra thinking I do that I shouldn'*t *takes all the brain energy away from actually important things"*. These responses support the construct distinction underlying *BodyThink*.

***BodyThink* Questionnaire Items**

The *BodyThink* questionnaire assesses cognitive complaints across seven cognitive domains. Participants rate the frequency of each difficulty on a four-point Likert scale: 0 (*never*), 1 (*sometimes*), 2 (*often*), 3 (*always*).

Items are presented in response to the prompt: "I generally..."

Full item composition and domain classifications are detailed in Supplementary Table S1.

*Study 2 BodyThink Items Ranked*

| **Rank** | **Item** | **Description** | **Domain** | **Often+always endorsement** |
| --- | --- | --- | --- | --- |
| 1 | 41 | Accepting compliments about my looks | Social cognition | 73.9% |
| 2 | 39 | Stopping myself from thinking negative thoughts | Executive function | 67.2% |
| 3 | 40 | Assume it's about me when I hear someone laughing/whispering | Social cognition | 64.0% |
| 4 | 26 | Make silly mistakes | Attention | 59.6% |
| 5 | 27 | Feel like I have brain fog | Processing | 59.6% |
| 6 | 28 | Thinking isn'*t* as good as it used to be | Processing | 59.6% |
| 7 | 10 | Am easily distracted | Attention | 58.9% |
| 8 | 7 | Take longer than normal to complete daily tasks | Processing | 58.7% |
| 9 | 1 | Remembering people's names | Memory | 57.3% |
| 10 | 24 | Being flexible | Executive function | 56.8% |
| 11 | 5 | Concentrating when reading a book | Attention | 56.4% |
| 12 | 11 | Doing simple mental calculations | Executive function | 55.7% |
| 13 | 2 | Finding objects of daily use | Memory | 55.0% |
| 14 | 23 | Get stuck and can'*t* figure out how to fix situations | Executive function | 54.7% |
| 15 | 13 | Learning new information | Memory | 54.3% |
| 16 | 31 | Accurately reading social situations | Social cognition | 54.3% |
| 17 | 9 | Finding the words to express my ideas | Language | 53.1% |
| 18 | 14 | Keeping focused for a long time | Attention | 52.9% |
| 19 | 22 | Flexibility in decision-making | Executive function | 52.4% |
| 20 | 20 | Finding words, forming sentences | Language | 50.3% |
| 21 | 15 | Remembering to do household chores | Memory | 50.3% |
| 22 | 12 | Following a conversation | Attention | 50.1% |
| 23 | 6 | Recalling what I have been told recently | Memory | 49.7% |
| 24 | 19 | Doing two things at once | Executive function | 49.7% |
| 25 | 25 | Paying attention to details | Attention | 49.7% |
| 26 | 30 | Seeing another person's perspective | Social cognition | 48.7% |
| 27 | 21 | Planning activities | Executive function | 48.3% |
| 28 | 4 | Placing important events in time | Memory | 48.0% |
| 29 | 29 | Understanding body language/facial expressions | Social cognition | 47.3% |
| 30 | 33 | Take things too literally/don'*t* get jokes | Social cognition | 45.5% |
| 31 | 3 | Remembering important situations | Memory | 44.6% |
| 32 | 38 | Forget where I parked my car | Memory | 43.4% |
| 33 | 36 | Being detail-oriented | Attention | 43.2% |
| 34 | 17 | Remembering important events | Memory | 42.7% |
| 35 | 35 | Noticing little differences | Attention | 42.0% |
| 36 | 16 | Remembering to take medications | Memory | 41.8% |
| 37 | 18 | Remembering the names of well-known people | Memory | 41.1% |
| 38 | 37 | Completing jigsaw puzzles | Visuospatial | 39.3% |
| 39 | 32 | Recognising people | Memory | 37.6% |
| 40 | 34 | Understanding sarcasm | Social cognition | 35.8% |
| 41 | 8 | Feel disoriented in the street | Attention | 35.3% |

*Note.* (*N* = 433) Response options were Never (0), Sometimes (1), Often (2), Always (3). All 41 *BodyThink* items ranked by frequency of endorsement (Often + Always).
